# Overexpression of denticleless E3 ubiquitin protein ligase homolog (DTL) is related to poor outcome in gastric carcinoma

**DOI:** 10.18632/oncotarget.5620

**Published:** 2015-10-13

**Authors:** Hiroki Kobayashi, Shuhei Komatsu, Daisuke Ichikawa, Tsutomu Kawaguchi, Shoji Hirajima, Mahito Miyamae, Wataru Okajima, Takuma Ohashi, Toshiyuki Kosuga, Hirotaka Konishi, Atsushi Shiozaki, Hitoshi Fujiwara, Kazuma Okamoto, Hitoshi Tsuda, Eigo Otsuji

**Affiliations:** ^1^ Division of Digestive Surgery, Department of Surgery, Kyoto Prefectural University of Medicine, Kawaramachihirokoji, Kamigyo-ku, Kyoto, Japan; ^2^ Department of Pathology, National Cancer Center Hospital, Tokyo, Japan; ^3^ Department of Basic Pathology, National Defense Medical College, Saitama, Japan

**Keywords:** gastric cancer, DTL, prognosis, oncogene, biomarker

## Abstract

**Background:**

Denticleless E3 ubiquitin protein ligase homolog (DTL) has been identified in amplified region (1q32) of several cancers and has an oncogenic function. In this study, we tested whether DTL acts as a cancer-promoting gene through its activation/overexpression in gastric cancer (GC).

**Methods:**

We analyzed 7 GC cell lines and 100 primary tumors that were curatively resected in our hospital between 2001 and 2003.

**Results:**

Overexpression of the DTL protein was detected in GC cell lines (4/7 cell lines; 57%) and primary GC tumor samples (42/100 cases; 42%). Knockdown of DTL using several specific siRNAs inhibited the proliferation, migration and invasion in a *TP53* mutation-independent manner. Overexpression of the DTL was significantly correlated with lymphatic invasion, deeper tumor depth and higher recurrence rate. Patients with DTL-overexpressing tumors had a worse survival rate than those with non-expressing tumors in overall survival (*P* = 0.0498, log-rank test) and disease-free survival (*P* = 0.0324, log-rank test). In a multivariate analysis, DTL positivity was independently associated with a worse overall survival (*P* = 0.0104, hazard ratio 3.7 [1.36–10.1]) and disease-free survival (*P* = 0.0070 (hazard ratio, 3.9 (1.45–10.46)) following radical gastrectomy.

**Conclusions:**

These findings suggest that DTL overexpression plays a crucial role in tumor cell proliferation and highlights its usefulness as a prognosticator and potential therapeutic target in gastric cancer.

## INTRODUCTION

Gastric cancer is the second leading cause of cancer-related death in the world [1]. Recent advances in diagnostic techniques and peri-operative management have increased the early detection of gastric cancer and decreased the mortality rate. However, patients with advanced disease still frequently develop recurrent disease after extended radical resections and consequently have extremely poor survival rates [2].

Numerous genes have been analyzed in an attempt to understand the molecular mechanisms and to improve clinical outcomes for human gastric cancers; however, only a few genes with frequent alterations have been identified [3]. Mutations of *TP53*, *APC* [4], and *E-cadherin* [5], amplification and overexpression of *MET* and *ERBB2* [6], oncogenic activation of *β-catenin* and *K-ras* [7, 8], inactivation of the mismatch repair gene *hMLH1* associated with microsatellite instability (MSI) [9], and hypermethylation of *p16* have been repeatedly reported [10, 11]. As discussed in these reports, studies have attempted to identify the biological factors involved in the malignant potential of gastric cancer. However, in clinical settings, only a few genes have been investigated as therapeutic targets and/or diagnostic biomarkers [12], suggesting that novel genes associated with the progression of gastric cancer need to be identified.

The DTL protein was cloned as a downregulated gene during retinoic acid-induced differentiation of the human embryonal carcinoma cell line NT2 [13]. Several studies have revealed that DTL has an oncogenic function in several types of cancer, such as hepatocellular carcinoma, breast cancer, and Ewing sarcoma [14–16]. Moreover, DTL plays a pivotal role in regulating the protein stability of p53 [17]. DTL was recently reported to have an oncogenic role in gastric carcinogenesis as established by *in vitro* analyses, and significantly overexpressed in gastric cancer tissues than their adjacent non-cancerous tissues [18]. However, to date, there has been no report on the clinical and prognostic significance of DTL overexpression in primary gastric cancer.

In this study, we show that DTL is frequently overexpressed in gastric cancer cell lines and primary gastric cancer tissues. Overexpression of DTL was a poor prognosticator independent of other prognostic factors. Additionally, we demonstrated that knockdown of DTL suppressed cell proliferation, migration, and invasion of DTL-overexpressing gastric cancer cells in a *TP53* mutation-independent manner. Our results provide evidence that DTL could be a promising clinical biomarker for determining malignant properties and a target for molecular therapy in patients with gastric cancer.

## RESULTS

### Overexpression of DTL in gastric cancer cell lines

**Figure 1:**
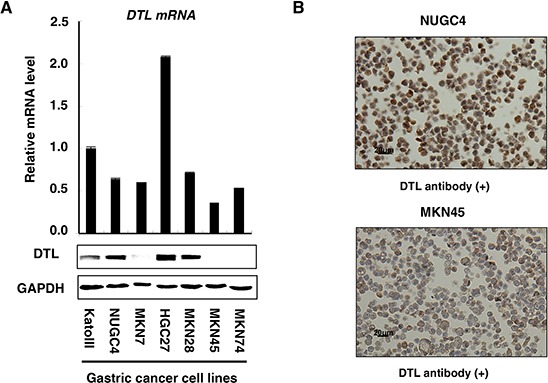
Expression profiles of DTL in 7 GC cell lines **A.** Quantitative real-time RT–PCR and western blotting analysis were performed using a DTL-specific antibody to determine DTL mRNA (top) and protein (bottom) expression in the gastric cancer cell lines KatoIII, NUGC4, MKN7, HGC27, MKN28, MKN45, and MKN74. DTL overexpression was observed in the KatoIII, NUGC4, HGC27, and MKN28 cells (4/7 lines, 57%). **B.** A formalin-fixed gastric cancer NUGC4 cell line that overexpresses DTL, in which > 50% of cells stained positively, was used as a positive control; an MKN45 cell line with a low expression of DTL was included as a negative control.

Western blotting analysis was performed using a DTL-specific antibody to determine DTL protein expression in the gastric cancer cell lines KatoIII, NUGC4, MKN7, HGC27, MKN28, MKN45, and MKN74 (Figure 1A). DTL overexpression was observed in the KatoIII, NUGC4, HGC27, and MKN28 cells (4/7 lines, 57%), suggesting that the DTL gene is a target for activation in gastric cancer cell lines. A formalin-fixed gastric cancer NUGC4 cell line DTL, in which >50% of cells showed staining, was used as a positive control, whereas a formalin-fixed gastric cancer MKN45 cell line with low expression of DTL and NUGC4 staining without the DTL antibody were included as a negative control (Figure 1B).

### Suppression of cell proliferation by downregulation of DTL expression

**Figure 2:**
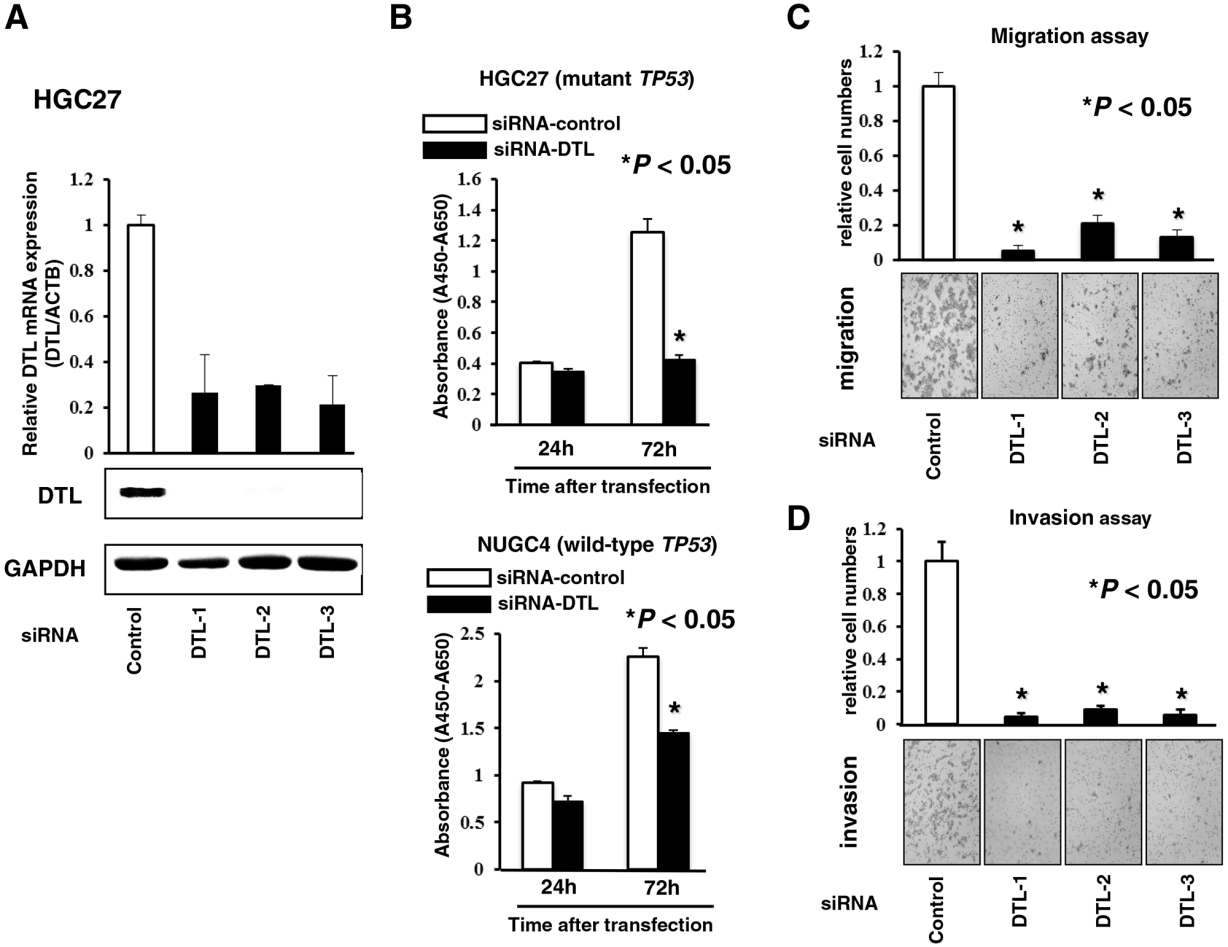
Effects of downregulation of DTL expression **A.** Loss-of-function screening was undertaken using three small interfering RNAs (siRNAs) targeting DTL in the HGC27 cell. The knockdown of a target DTL gene was confirmed by quantitative real-time RT–PCR (top) and western blotting (bottom). **B.** To assess cell growth, the numbers of viable cells at various time points after transfection were assessed by the colorimetric water-soluble tetrazolium salt (WST) assay in the HGC27 (mutant *TP53*) and NUGC4 (wild-type *TP53*) cell lines. **C.** Transwell migration (upper) and invasion (lower) assays using three siRNAs targeting DTL.

To gain insight into the potential role of DTL as an oncogene whose overexpression could be associated with gastric carcinogenesis, we first performed a cell proliferation assay using three siRNAs specific to DTL to investigate whether knockdown of DTL expression could suppress the proliferation of gastric cancer cells showing overexpression of the gene (Figure 2A). We performed cell proliferation assays using the HGC27 cell line, which has mutant *TP53*, and the NUGC4 cell line, which has wild-type *TP53*, because wild-type p53 is expected to be a more suitable substrate than mutant p53 for DTL [15–17]. The proliferation of both HGC27 and NUGC4 cells was significantly lower than controls at 72 h after transfection (Figure 2B). The proliferation inhibition rate of HGC27 and NUGC4 cells transfected with siRNA-DTL was 66.3% and 35.9%, respectively, suggesting that this inhibitory effect is not associated with *TP53* mutation status.

### Suppression of cell migration and invasion by knockdown of DTL

Transwell migration and invasion assays were performed to examine the invasive potential of both HGC27 and NUGC4 cells transfected with siRNA-DTL to migrate under different conditions. An uncoated membrane was used for migration assays, whereas a matrigel-coated membrane was used for invasion assays. The number of cells that migrated into the lower chamber was significantly lower for siRNA-DTL-transfected cells than for siRNA-control-transfected cells under both conditions (Figure 2C), suggesting that DTL may increase the ability of gastric cancer cells to migrate and invade.

### Immunohistochemical analysis of DTL expression in the primary gastric cancer tumors

**Figure 3:**
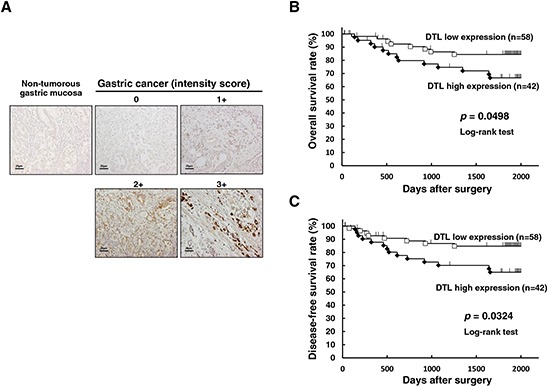
Immunohistochemical-staining analyses and postoperative overall survival curve according to the expression of DTL **A.** Specific immunostaining of the DTL protein in primary samples was confirmed. Expression of the DTL protein was observed in both the cytoplasm and nucleus of cancer cells. For scoring DTL expression, the intensity score was defined as 0 = negative, 1 = weak, 2= moderate, 3 = strong. The DTL high expression group had a significantly poorer prognosis than the low expression group in overall survival (*P* = 0.0498, log-rank test) **B.** and disease-free survival (*P* = 0.0324, log-rank test) **C.**

Because DTL protein was overexpressed in some gastric cancer cell lines, it was hypothesized that DTL was also highly expressed in gastric cancer tissues, playing a role in carcinogenesis and malignant outcome. We examined the clinicopathological significance of DTL expression in primary tumor samples of gastric cancer based on the immunohistochemical staining pattern of this protein. Specific immunostaining of the DTL protein in primary samples was confirmed using gastric cancer cell lines as positive (NUGC4) or negative controls (MKN45) (Figure 1B). Expression of the DTL protein was observed in both the cytoplasm and nucleus of cancer cells. In primary cases, DTL protein expression was negative in most of the non-tumorous gastric mucosal cell population (intensity score 0). We divided 100 gastric cancer tumors into a high expression group with both intensity scores ≥ 2 and ≥ 10% of tumor cells showing immunopositivity (*n* = 42 (42%)) and a low expression group in intensity scores ≤ 1 and/or < 10% of tumor cells showing immunopositivity (*n* = 58 (58%)) according to the intensity of DTL staining among tumor cells (Figure 3A). The high expression group had a significantly poorer prognosis than the low expression group for overall survival (*P* = 0.0498, log-rank test) (Figure 3B) and disease-free survival (*P* = 0.0324, log-rank test) (Figure 3C). Five-year overall survival rates of patients with DTL high- and low-expression cancer in each stage were 95% vs. 100% (*p* = 0.16) in stage I, 83% vs. 67% (*p*= 0.15) in stage II and 35% vs. 54% (*p* = 0.22) in stage III, respectively.

### Association between DTL protein levels and clinicopathological characteristics in primary gastric cancer

The relationship between the expression of the DTL protein and clinicopathological characteristics is summarized in Table 1. The protein expression of DTL was significantly associated with lymphatic invasion (*P* = 0.0137), deeper depth of invasion (*P* = 0.0256), and recurrence rate (*P* = 0.0199), whereas other characteristics, including histological grade, were not. In the Cox proportional hazard regression model (Table 2), univariate analyses demonstrated that DTL protein expression, macroscopic appearance, venous invasion, lymphatic invasion, pT category, pN category, and tumor size were significantly associated with cause-specific survival. When the data were stratified for multivariate analysis using both forward and backward stepwise Cox regression procedures, DTL immunoreactivity in tumor cells remained significant at *P* = 0.0104 (hazard ratio, 3.7 (1.36–10.11)) for overall survival (Table 2) and *P* = 0.0070 (hazard ratio, 3.9 (1.45–10.46)) for disease-free survival (Supplementary Table S1) in all patients, suggesting that DTL immunoreactivity can be an independent predictor of overall survival.

**Table 1:** Association between clinicopathologic characteristics and DTL expression

	*n*	DTL immunoreactivity		*P* value*
		high expression	low expression	
Total	100	42 (42%)	58 (58%)	
Gender				
Male	59	29 (69%)	30 (52%)	0.0821
Female	41	13 (31%)	28 (48%)	
Age (y)				
Mean 61.22 (range:28–87)				
<60	40	16 (38%)	24 (41%)	0.7407
≥60	60	26 (62%)	34 (59%)	
Histopathological grading				
Differentiated	45	20 (48%)	25 (43%)	0.6542
Undifferentiated	55	22 (52%)	33 (57%)	
Tumor size (mm)				
< 25	32	12 (28%)	20 (34%)	0.5317
≥25	68	30 (72%)	38 (66%)	
Venous invasion				
v0	72	27 (64%)	45 (78%)	0.1462
v1–2	25	15 (36%)	10 (17%)	
v3	3	0 (0%)	3 (5%)	
Lymphatic invasion				
ly0	55	16 (38%)	39 (67%)	**0.0137**
ly1–2	39	24 (57%)	15 (26%)	
ly3	6	2 (5%)	4 (7%)	
TNM classification				
pT categories				
pT1–3	78	28 (67%)	50 (81%)	**0.0199**
pT4	22	14 (33%)	8 (19%)	
pN categories				
pN0–2	84	33 (79%)	51 (86%)	0.2076
pN3	16	9 (21%)	7 (14%)	
Recurrennce				
Absent	78	28 (67%)	50 (81%)	**0.0199**
Present	22	14 (33%)	8 (19%)	

NOTE. Significant values are in bold type.

* *P* values are from χ^2^ or Fisher's exact test and were significant at <0.05.

**Table 2:** Multivariate analysis for overall survival using stepwise Cox regression procedures

Variables	Univariate^a^			Multivariate^b^		
	*P* value	HR^c^		95%CI^d^		*P* value
Gender						
male *versus* female	0.8990			–		
Age						
≥60 *versus* <60	0.7325			–		
Histological type						
Undiffe.versus Diffe.	0.7982			–		
Tumor size (mm)						
≥25 *versus* <25	< **0.0005**			–		
Venous invasion						
v2–3 *versus* v0–1	< **0.005**			–		
Lymphatic invasion						
ly2–3 *versus* ly0–1	< **0.0001**	12.74	4.045	–	40.05	< **0.0001**
pT-stage						
T4 *versus* T1–3	< **0.0001**			–		
pN-stage						
N3 *versus* N0–2	**<0.0001**	7.591	2.661	–	21.65	**0.0002**
DTL expression						
high *versus* low	**0.0499**	3.709	1.360	–	10.11	**0.0104**

a Kaplan and Meier method, and the statistical significance was determined by log-rank test

b Multivariate survival analysis was performed using Cox's proportional hazard model

c HR:hazard ratio

d CI:confidence interval

## DISCUSSION

The *DTL* gene, which is also known as the *CDT2/RAMP/DCAF2/L2DTL* gene, is located on chromosome 1q32; this area is frequently amplified in human solid cancers [19]. *DTL* encodes a putative 730 amino acid protein that contains six highly conserved five WD40-repeat domains and physically interacts with the DDB1/CULLIN4 complex (CRL4). Cullin-RING ubiquitin ligases (CRLs) are the largest family of E3 ligase. DTL is a substrate-specific adapter of CRL4 (CRL4^DTL^) and mediates the polyubiquitination and subsequent degradation of CDT1 [20–22], p12 [23], p21 [24–26], SET8 [27–29] and Gcn5 [30]. In addition, CRL4^DTL^ was shown to promote degradation in some of these substrates, such as CDT1 [20], p21 [24], and SET8 [27]_,_ by interaction with PCNA. These molecular mechanisms indicate that DTL has a crucial physiological role. Moreover, DTL has been identified as a clinically relevant factor for enhanced metastatic potential in HCC [14] and tumor growth of breast cancer cells [15] and is highly proliferative in Ewing sarcoma [16]. These findings prompted us to determine the clinicopathological and prognostic significance of DTL overexpression/activation in primary gastric cancer. However, to date, there has been no report on the clinical significance of DTL in patients with primary gastric cancer.

In this study, we hypothesized that the overexpression/activation of DTL may promote tumor cell proliferation and/or survival in gastric cancer cells. To test this hypothesis, we examined the expression status of DTL and the clinicopathological parameters, as well as the biological significance of its expression in gastric cancer cell lines and primary tumor tissues of gastric cancer. We demonstrated that DTL was overexpressed in 48% (29/60) of primary gastric cancer tissues and 57% (4/7) of gastric cancer cell lines and that this overexpression was a poor prognosticator independent of other prognostic factors. The prognosis of gastric cancer patients was associated with the intensity of DTL activity. In addition, downregulation of DTL expression suppressed cell proliferation, migration, and invasion in gastric cancer cell lines.

While investigating the interaction between DTL and p53, Banks *et al*. reported that DTL and PCNA regulate p53 polyubiquitination, and the inactivation of DTL induces p53 stabilization and cell growth arrest [17]. Li *et al*. reported that DTL-mediated apoptosis in gastric cancer cells is dependent on the p53 pathway [18]. Pan *et al*. reported that DTL overexpression was associated with poor prognosis, particularly in *TP53*-mutated hepatocellular carcinoma cell lines [14]. In our study, knockdown using several siRNAs specific to DTL in DTL-expressing gastric cancer cells significantly reduced cell proliferation in a *TP53* mutation-independent manner. In addition, migration and invasion were inhibited.

Our results indicate that DTL plays a crucial role in tumor cell proliferation, migration and invasion through a p53-dependent or independent pathway.

In conclusion, this is the first report demonstrating that DTL has a pivotal oncogenic role independent of TP53 mutation and is an independent poor prognostic factor in gastric cancer. Although studies of larger cohorts are needed to validate these findings before moving to a clinical setting, our results suggest that DTL is an important molecular marker for determining malignant properties and targets for molecular therapy in patients with this lethal disease.

## MATERIALS AND METHODS

### Gastric cancer cell lines and primary tissue samples

Seven gastric cancer cell lines, KatoIII, NUGC4, HGC27, MKN7, MKN28, MKN45 and MKN74, were used in these studies. HGC27 cells were cultured in Dulbecco's Minimum Essential Medium (DMEM): F12 medium, and the other cells were cultured in Roswell Park Memorial Institute (RPMI)-1640 medium (Sigma, St. Louis, MO). All media were purchased from Sigma and supplemented with 100 mL/L FBS (Trace Scientific, Melbourne, Australia). All cell lines were cultured in 50 mL/L carbon dioxide at 37°C in a humidified chamber. Primary gastric cancer tumor samples were obtained from 100 consecutive gastric cancer patients who underwent curative gastrectomy (R0) at the Division of Digestive Surgery, Department of Surgery, Kyoto Prefectural University of Medicine (Kyoto, Japan) between 2001 and 2003 and were embedded in paraffin after 24 h of formalin fixation. Relevant clinical and survival data were available for all patients. Written consent was always obtained in a formal style and after approval by the local Ethics Committee. None of the patients underwent endoscopic mucosal resection, palliative resection, preoperative chemotherapy, or radiotherapy, and none of them had synchronous or metachronous multiple cancers in other organs. Disease stage was defined in accordance with the International Union against Cancer tumor-lymph node-metastases (TNM) classification (7th edition) [31]. The median follow-up period for surviving patients was 105.1 months (ranging from 0.8 to 163.5 months).

### Quantitative real-time RT–PCR

Single-stranded complementary DNAs generated from total RNA were amplified with primers specific for each gene. Levels of messenger RNA (mRNA) expression were measured by quantitative real-time fluorescence detection (ABI StepOnePlus™ Sequence Detection System; Applied Biosystems, Foster City, CA) using TaqMan Gene Expression Assays (Hs00978565_m1 for DTL; Applied Biosystems) according to the manufacturer's instructions. The results of gene expression were calculated as the ratio between DTL and an internal reference gene (Hs99999903_m1 for β-actin; Applied Biosystems) that provides a normalization factor for the amount of RNA isolated from a specimen. The ratio was subsequently normalized to the KatoIII cell line (relative expression). This assay was performed in duplicate for each sample.

### Western blotting

Anti-DTL rabbit polyclonal antibody (NB100–40840) was purchased from Novus Biologicals, LLC (Colorado, USA) and anti-GAPDH antibody was purchased from Santa Cruz Biotechnology (Santa Cruz, CA). DTL antibody is an affinity purified rabbit polyclonal antibody raised against the recombinant peptide containing a portion of the DTL protein with a His-tag at its C-terminus. The cells were lysed, and their proteins were extracted using the M-PER^®^ Mammalian Protein Extraction Reagent (Thermo Scientific, USA).

### Loss-of-function by small interfering RNA (siRNA) and cell growth analysis

For knocking down endogenous DTL expression, each of the small interfering RNAs (siRNA) targeting DTL (Stealth RNAi™, siRNA-DTL-1; # HSS122293, siRNA-DTL-2; # HSS122295 and siRNA-DTL-3; # HSS182090; Invitrogen, Carlsbad, CA) and the control were transfected into cells (10 nmol/l) using Lipofectamine RNAiMAX (Invitrogen, Carlsbad, CA) according to the manufacturer's instructions. The knockdown of a target gene was confirmed by quantitative real-time RT–PCR and western blotting. To measure cell proliferation, the number of viable cells 24 and 72 h after siRNA transfection was assessed by the colorimetric water-soluble tetrazolium salt (WST) assay (Cell counting kit-8; Dojindo Laboratories, Kumamoto, Japan) [32].

### Transwell migration and invasion assays

Transwell migration and invasion assays were carried out in 24-well modified Boyden chambers (transwell-chamber, BD Transduction, Franklin Lakes, NJ). The upper surface of the 6.4 mm diameter filters with 8-μm pores was pre-coated with (invasion assay) or without (migration assay) Matrigel (BD Transduction). The siRNA transfectants (2 × 10^4^ cells per well) were transferred into the upper chamber. Following 48 h of incubation, migrated or invasive cells on the lower surface of filters were fixed and stained with the Diff-Quik stain (Sysmex, Kobe, Japan), and stained cell nuclei were counted directly in triplicate. We assessed the invasive potential by calculating the ratio of the percentage invasion through the Matrigel-coated filters relative to migration through the uncoated filters of test cells over that in the control counterparts [33, 34].

### Immunohistochemistry

Anti-DTL rabbit polyclonal antibody (NB100–40840), which was the same antibody as western blotting analysis and was purchased from Novus Biologicals, LLC (Colorado, USA), was used. Tumor samples were fixed with 10% formaldehyde in PBS, embedded in paraffin, sectioned into 5-μm-thick slices, and subjected to immunohistochemical staining of the DTL protein with the avidin-biotin-peroxidase method as described by Naoi *et al*. [35]. In brief, after deparaffinization, endogenous peroxidases were quenched by incubating the sections for 30 min in 3% H_2_O_2_. Antigen retrieval was performed by heating the samples in 10 mmol/L citrate buffer (pH 6.0) at 95°C for 60 min. After treatment with Block Ace (Dainippon Sumitomo Pharmaceutical, Osaka, Japan) for 20 min at room temperature, sections were incubated at 4°C overnight with an anti-DTL (1:1000) antibody. The avidin-biotin-peroxidase complex system (Vectastain Elite ABC universal kit; Vector Laboratories Inc., Burlingame, CA) was used for color development with diaminobenzidine tetrahydrochloride. The slides were counterstained with Mayer's hematoxylin. A formalin-fixed gastric cancer NUGC4 cell line that overexpresses DTL, in which > 50% of cells stained positive, was used as a positive control, whereas a formalin-fixed gastric cancer MKN45 cell line with low expression of DTL was included as a negative control.

To evaluate DTL expression, in the percentage of tumor cells showing DTL immunopositivity, primary tumors with at least 10% or more of the total cell population was judged positive, and less than 10% was judged negative. In the intensity of DTL expression, the intensity score (0 = negative, 1 = weak, 2 = moderate, 3 = strong) was examined. Namely, primary tumors with non-detectable DTL expression, which was similar to non-tumorous gastric mucosa and stroma, were given an intensity score of 0, whereas those with the greatest DTL abundance were given an intensity score of 3. The remaining tumors were categorized with intensity scores of 1 or 2 according to the intensity of immunohistochemical staining for DTL. The expression of DTL was regarded as high expression in both intensity scores ≥ 2 and ≥ 10% of tumor cells showing immunopositivity or low expression in intensity scores ≤ 1 and/or < 10% using high-powered (x 200) microscopy [36, 37].

### Statistical analysis

Clinicopathological variables pertaining to the corresponding patients were analyzed for statistical significance using the chi-squared test or Fisher's exact test. For the analysis of survival, Kaplan–Meier survival curves were constructed for groups based on univariate predictors, and differences between the groups were analyzed with the log-rank test. Univariate and multivariate survival analyses were performed using the likelihood ratio test of the stratified Cox proportional hazards model. Differences between subgroups were tested with the non-parametric Mann–Whitney *U*-test. Differences were assessed with a two-sided test and were considered statistically significant at *P* < 0.05.

## SUPPLEMENTARY MATERIALS TABLE


