# Supplementary material for: Overexpression of denticleless E3 ubiquitin protein ligase homolog (DTL) is related to poor outcome in gastric carcinoma
Source: Oncotarget. 2015 Oct 13;6(34):36615–24. doi: 10.18632/oncotarget.5620 (PMC4742199; doi:10.18632/oncotarget.5620)
Supplement: Supplementary file 1 [file oncotarget-06-36615-s001.pdf]

SUPPLEMENTARY TABLE

Supplementary Table S1: Multivariate analysis for disease-free survival using stepwise Cox regression procedures

Variables	Univariate[a]	Multivariate[b]				
	P value	HR[c]	95%CI[d]			*P* value
Gender						
male *versus* female	0.6723			–		
Age						
≥60 *versus* < 60	0.1342			–		
Histological type						
Undiffe.versus Diffe.	0.9462			–		
Tumor size (mm)						
≥25 *versus* < 25	**<0.0005**			–		
Venous invasion						
v2–3 *versus* v0–1	**<0.005**	3.179	1.081	–	9.348	**0.0356**
Lymphatic invasion						
ly2–3 *versus* ly0–1	**<0.0001**	12.73	4.012	–	40.40	**<0.0001**
pT-stage						
T4 *versus* T1–3	**<0.0001**			–		
pN-stage						
N3 *versus* N0–2	**<0.0001**	6.969	2.488	–	19.53	**0.0002**
DTL expression						
high *versus* low	**0.0324**	3.897	1.451	–	10.46	**0.0070**

[a]Kaplan and Meier method, and the statistical significance was determined by log-rank test
[b]Multivariate survival analysis was performed using Cox's proportional hazard model
[c]HR:hazard ratio
[d]CI:confidence interval
